# Prenatal ethanol exposure causes anxiety-like phenotype and alters synaptic nitric oxide and endocannabinoid signaling in dorsal raphe nucleus of adult male rats

**DOI:** 10.1038/s41398-022-02210-7

**Published:** 2022-10-10

**Authors:** Saida Oubraim, Ruixiang Wang, Kathryn Hausknecht, Martin Kaczocha, Roh-Yu Shen, Samir Haj-Dahmane

**Affiliations:** 1 Department of Pharmacology and Toxicology, State University of New York, 1021 Main Street, Buffalo, NY 14203 USA; 2 Department of Anesthesiology, Stony Brook University, Stony Brook, NY 11794 USA; 3 University at Buffalo Neuroscience Program, Jacobs School of Medicine and Biomedical Sciences, University at Buffalo, State University of New York, 1021 Main Street, Buffalo, NY 14203 USA

**Keywords:** Neuroscience, Physiology

## Abstract

Mood disorders, including anxiety and depression caused by prenatal ethanol exposure (PE) are prevalent conditions in fetal alcohol spectrum disorders (FASDs). Prenatal ethanol exposure is associated with persistent dysfunctions of several neurotransmitter systems, including the serotonin (5-HT) system, which plays a major role in mood regulation and stress homeostasis. While PE is known to disrupt the development of the 5-HT system, the cellular mechanisms by which it alters the function of dorsal raphe nucleus (DRn) 5-HT neurons and their synaptic inputs remain unknown. Here, we used a second-trimester binge-drinking pattern PE (two daily gavages of 15% w/v ethanol at 3 g/kg, 5–6 h apart) during gestational days 8 - 20 and measured anxiety-like behaviors of adult male rats using the elevated plus (EPM) and zero (ZM) mazes. We also employed ex-vivo electrophysiological and pharmacological approaches to unravel the mechanisms by which PE alters the excitability and synaptic transmission onto DRn 5-HT neurons. We found that PE enhanced anxiety-like behaviors in adult male rats and induced a persistent activation of DRn 5-HT neurons. The PE-induced activation of DRn 5-HT neurons was largely mediated by potentiation of DRn glutamate synapses, which was caused by activation of the nitrergic system and impaired endocannabinoid signaling. As such, the present study reveals “push-pull” effects of PE on nitrergic and eCB signaling, respectively, which mediate the enhanced activity of DRn 5-HT neurons and could contribute to anxiety-like behaviors observed in animal model of FASD.

## Introduction

Prenatal ethanol exposure (PE) profoundly alters brain development and causes an array of behavioral and cognitive deficits commonly referred to as Fetal Alcohol Spectrum Disorders (FASD) [1]. The major deficits of FASD include learning and memory deficits [2–5], attention deficit hyperactivity disorder [ADHD) [6, 7], and enhanced addiction propensity [8, 9]. Individuals with FASD also suffer from emotional disorders, including anxiety and depression [10, 11]. Similarly, animal studies have shown that PE causes anxiety and depression-like behaviors in adult rats [12, 13], thereby demonstrating that PE is a major risk factor for mood disorders.

Over the last decades, preclinical and clinical studies have revealed that emotional dysregulations induced by PE involve persistent alterations of several neurotransmitter systems [14], including the serotonin (5-HT) system. Serotonin neurons of the dorsal raphe nucleus (DRn), which provide the bulk of 5-HT in the brain [15] develop early during embryogenesis (E11-E15) [16] and are vulnerable to the teratogenic effects of ethanol. Indeed, animal studies have reported that PE reduces the development of 5-HT neurons [17, 18], decreases 5-HT projections [19, 20], reduces tryptophan hydroxylase type 2 (Trph2) [21] and 5-HT levels in the brain [17, 22]. These developmental alterations contribute, at least in part, to anxiety-like behaviors in PE rats [23]. Similarly, studies of FASD children have shown structural alterations of 5-HT projections and disrupted 5-HT neurotransmission, which correlate with the severity of behavioral deficits [24, 25]. These studies have led to the notion that alterations of 5-HT system play a prominent role in emotional dysregulations associated with FASD. Consequently, drugs that target central 5-HT transmission are commonly used to alleviate mood disorders in FASD children [26].

Although it is well documented that PE negatively impacts the development of 5-HT system, the mechanisms by which PE alters DRn 5-HT neurons function remain unknown. In the present study, we examined the impact of PE on anxiety-like behaviors, and investigated the cellular mechanisms by which PE alter the excitability of 5-HT neurons and their glutamate synapses.

## Materials and Methods

### Animal use

All experiments and procedures used in this study were approved by the University at Buffalo Animal Care and Use Committee in accordance with the National Institutes of Health Guideline for the Care and Use of Laboratory Animals. Animal breeding and PE treatment were performed as previously described [27]. The electrophysiological, behavioral and biochemical studies were conducted in distinct cohorts of control and PE male rats. To mitigate the potential litter effects, rats from different litters (2 rats/litter) were used for each experiments.

### Behavioral testing

Male rats (8–10 weeks) underwent two different behavioral tests: Elevated plus maze and zero maze as described previously [27]. The plus maze videos were manually coded and zero maze videos were analyzed with the ANY-maze 6.3 software (Stoelting Co., Wood Dale, IL, USA).

### Brain slice electrophysiology

Brain slices containing the DRn were obtained from control and PE rats (8 to 10 weeks) as previously described [28]. Following recovery at room temperature for at least 1 h, slices were transferred to a recording chamber (Warner Instruments, Hamden, CT, USA) mounted on a fixed upright microscope and continuously perfused (2 to 3 ml/min) with artificial cerebrospinal fluid (ACSF) of the following composition (in mM): 119 NaCl; 2.5 KCl; 2.5 CaCl_2_; 1.3 MgSO_4_; 1 NaH_2_PO_4_; 26.2 NaHCO_3_; 11 glucose and continuously bubbled with a mixture of 95% O_2_/5% CO_2_ at 30 ± 1°C. Somatic cell-attached and whole-cell recordings were performed from dorsal medial (dmDRn) and ventral medial (vmDRn) subdivisions of the DRn using potassium-gluconate based intracellular solution (in mM): 120 potassium gluconate; 10 KCl, 10 Na_2_ -phosphocreatine, 10 HEPES, 1 MgCl_2_, 1 EGTA, 2Na_2_-ATP, and 0.25 Na-GTP; pH 7.3; osmolality 280–290 mOsm/kg. Serotonin neurons were identified by their unique electrical properties, including slow firing activity, large after-hyperpolarization and 5-HT_1A_ receptor-induced membrane hyperpolarization [28, 29]. Excitatory postsynaptic currents (EPSCs) were evoked by single square-pulses (duration = 100–200 μs) delivered at 0.1 Hz. To access paired-pulse ratio (PPR), a pair of EPSCs were evoked with an inter-stimulus interval of 50 ms. The intensity of the stimulus was adjusted to evoke 75 % of the maximal amplitude of EPSCs. α-amino-3-hydroxy-5-methyl-4- isoxazolepropionic acid (AMPA) receptor-mediated EPSCs were recorded from neurons voltage clamped at −70 mV in the presence of a GABA_A_ receptor antagonist picrotoxin (100 μM). The cell input resistance and access resistance (10 – 20 mΩ) were monitored throughout the experiment using 5 mV hyperpolarizing voltage steps (500 ms duration). Recordings were discarded when the input and series resistance changed by more than 10–20%. To isolate miniature action potential independent EPSCs (mEPSCs), tetrodotoxin (TTX, 1 µM) was added to Ringer’s solution in the bath. All data were acquired using the pClamp 10.2 software (Molecular Devices, Union City, CA, USA).

### Endocannabinoids levels quantification

The levels of 2-arachydonyl glycerol (2-AG) and anandamide (AEA) in rat DRn slices were quantified using mass spectrometry and deuterated internal 2-AG and AEA standards as described previously [30, 31].

### Quantitative polymerase-chain reaction

RNA was extracted from brainstem slices using the RNeasy mini kit (Qiagen) and cDNA synthesis was carried out using the SuperScript III First Strand synthesis system (ThermoFisher). qPCR was performed using PowerUp SYBR green (ThermoFisher) on a StepOnePlus real-time PCR system (Applied Biosystems). The following primers were used: CB1R, Forward: AAGTCGATCCTAGATGGCCTT and Reverse: TCCTAATTTGGATGCCATGTCTC; Actin, Forward: GAGGCTCTCTTCCAGCCTTC and Reverse: CGGATGTCAACGTCACACTT. Quantification was performed using the 2-ΔΔCt method with actin serving as the housekeeping gene. Control reactions lacking template cDNA were included in each run. qPCR data were analyzed by comparing ΔCt values using an unpaired T-test.

### Data analysis

Evoked excitatory postsynaptic currents (eEPSCs) were analyzed using Clampfit 10.2 software (Molecular Devices, Union City, CA, USA). The amplitude of eEPSCs is determined by measuring the average current during a 2 ms at the peak of each eEPSC, subtracted from the baseline current and normalized to the mean baseline amplitude recorded for at least 5 min prior drug application. The PPRs (eEPSC_2_/eEPSC_1_) were averaged for at least 60 trials. Miniature excitatory postsynaptic currents (mEPSCs) were analyzed with Mini Analysis Software (Synaptosoft, Decatur, GA). The synaptic events were selected using amplitude threshold (5 pA), rise time (1 ms), and area threshold (30 fC). All selected events were further visually inspected to prevent noise from compromising the analysis. The decay kinetic of mEPSCs was fitted with mono-exponential decay of the following equation: y = A_0_ + Ae^kt^, where A_0_ is the baseline current, A is the peak current and k is the decay time constant. The data distribution was tested for normality using Shapiro–Wilk test. Parametric (independent and paired t-tests), and non-parametric tests (Mann-Whitney test) were used for normally and not normally distributed data, respectively. For comparison between groups, the independent t-test and analysis of variance (ANOVA) using post hoc Tukey tests were used. For mEPSC comparison, the Kolmogorov–Smirnov tests (K–S tests) were used. Statistical analysis was performed with Origin 9.0 software (MicroCal Software Inc., Northampton, MA, United States) and significance was set at α = 0.05. The results in the text and figures are expressed as mean ± SEM.

### Drug and chemicals

Chemicals and salts were obtained from Fisher Scientific (Pittsburgh, PA, USA). N-(Piperidin-1-yl)-5-(4-iodophenyl)-1-(2,4-dichlorophenyl)-4-methyl-1H-pyrazole-3-carboxamide (AM 251), Picrotoxin purchased from Tocris Biosciences (Minneapolis, MN, USA), S-Nitroso-N-acetyl-DL-penicillamine (SNAP), Nω-Nitro-L-arginine methyl ester hydrochloride (L-NAME), 2-(4-Carboxyphenyl)-4,4,5,5-tetramethylimidazoline-1-oxyl-3-oxide (carboxy-PTIO) and 8-(4-Chloorophenylthio)-guanosine 3’, 5’-cyclic monophosphate (8 pCPT-cGMP) were purchased from Sigma-Aldrich (Saint Louis, MO, USA).

## Results

### Prenatal ethanol exposure causes anxiety-like behaviors in adult rats

**Fig. 1:**
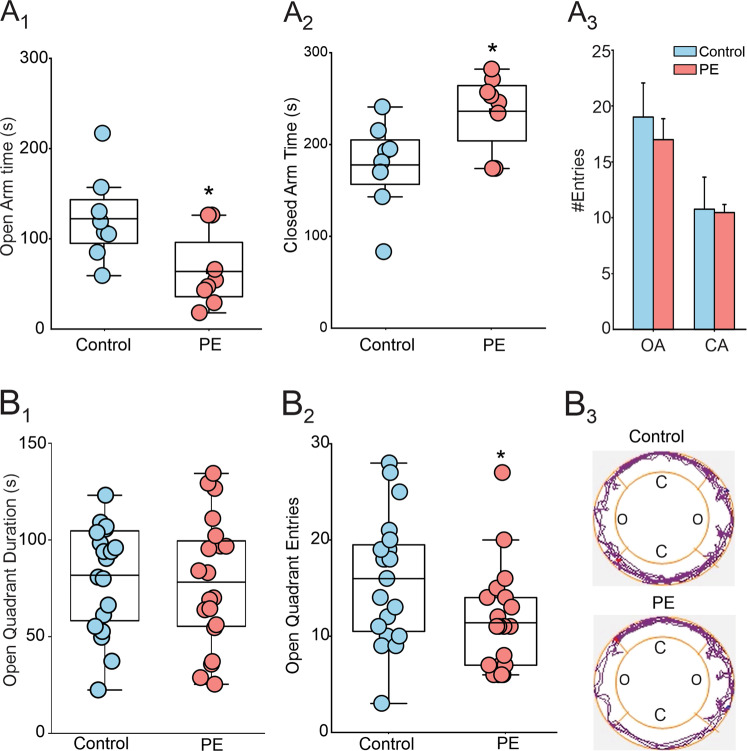
Prenatal ethanol exposure causes anxiety phenotype in adult rats. **A** PE increases anxiety-like behavior in the elevated plus maze test. **A**_**1**_ Average duration of stay in open arms of control () and PE rats () (*N* = 8 from 4 litters, *p* < 0.05). **A**_**2**_ Average duration of stay in closed arms (*N* = 8 from 4 litters, *p* < 0.05). **A**_**3**_ Summary graph of the average entries of control and PE rats to open and closed arms. **B** PE increases anxiety-like behavior in the zero-maze test. **B**_**1**_ Average duration of stay in open quadrants of control and PE rats (*N* = 20 from 5 litters, *p* > 0.05). **B**_**2**_ In the elevated zero maze test, PE rats made fewer entries into the open quadrants (*N* = 20 from 5 litters, *p* < 0.05). **B**_**3**_ Representative tracking plots obtained from control (upper panel) and PE (lower panel) rats in the zero-maze test, which shows that compared with the control rat, the PE rat travelled mostly on quadrants that they were initially placed on, reluctant to venture out to the farther quadrants.*: *p* < 0.05, independent-samples t-tests, N number of rats.

The impact of PE on anxiety-like behaviors was tested in the elevated plus maze (EPM) and zero maze tests (ZM). In the EPM test, compared to control, PE rats spent less time in open arms (Control: 122.4 ± 17 s; PE: 63.63 ± 14.55 s; Fig. 1A_1_), and more time in closed arms (control: 180.5 ± 17.9 s; PE: 245.6 ± 18.5 s; Fig. 1A_2_), with no significant difference in open arms (control: 19.0 ± 3.08; PE: 17.00 ± 1.87; Fig. 1A_3_), and closed arms entries (control: 10.75 ± 2.87; PE: 10.75 ± 0.75; Fig. 1A_3_), indicating that PE increases anxiety-like behaviors without altering locomotor activity. In ZM test, no differences in open quadrant time were observed (control: 81.74 ± 6.17 s, *n* = 20; PE = 78.22 ± 7.45 s; Fig. 1B_1_) between cohort. However, PE rats made fewer entries into open quadrants than control (control: 16.00 ± 1.46; PE: 11.40 ± 1.2; Fig. 1B_2_) and spent more time on quadrants they were initially on, reluctant to venture toward farther quadrants, as shown by the representative tracking plots (Fig. 1B_3_). Collectively, these results indicate that PE increases anxiety-like behavior in adult rats.

### Prenatal ethanol exposure enhances the electrical activity of putative DRn 5-HT neurons

**Fig. 2:**
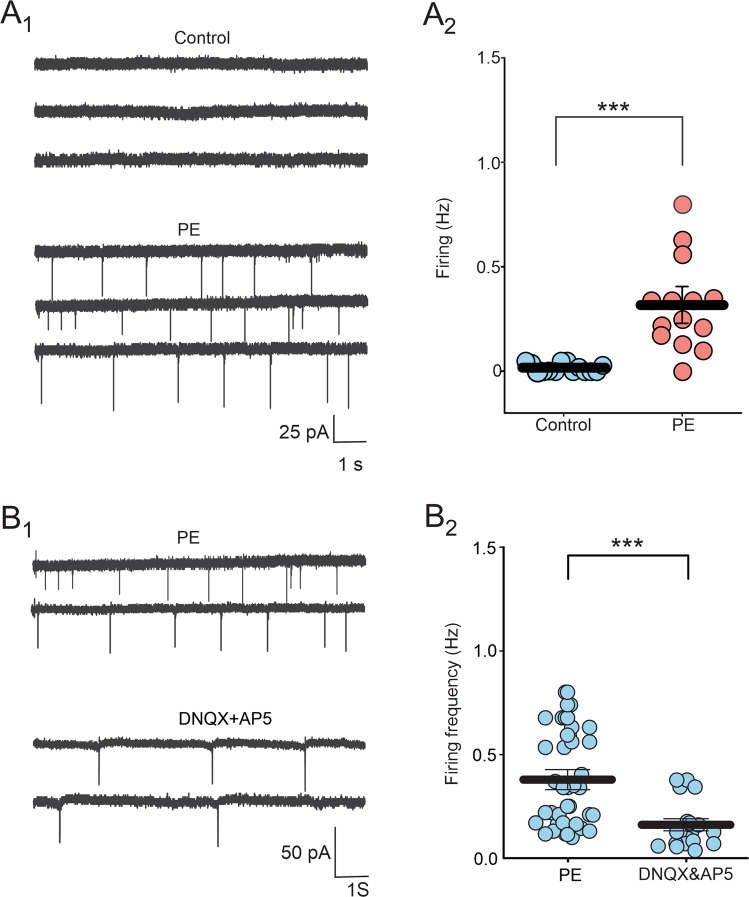
Prenatal ethanol exposure increases the spontaneous firing activity of DRn 5-HT neurons by increasing glutamatergic synaptic transmission. **A** PE increases the firing activity of DRn 5-HT neurons. **A**_**1**_ Cell-attached recordings of DRn 5-HT neurons from control (upper panel) and PE rats (lower panel). **A**_**2**_ Summary of the average firing frequency of DRn 5-HT neurons recorded from control (, *n* = 14, *N* = 5 from 3 litters) and PE rats (, *n* = 14, *N* = 5 from 3 litters, Mann-Whitney test: *p* < 0.001). **B** Blockade of ionotropic glutamate receptors significantly reverses the PE-induced increase in spontaneous firing activity of DRn 5-HT neurons. **B**_**1**_ Recordings of spontaneous firing activity of DRn 5-HT neurons from PE rats in the absence (upper traces) and presence (lower traces) of AMPA and NMDA receptor antagonists, DNQX (20 µM) and AP5 (20 µM), respectively. **B**_**2**_ Summary graph of the spontaneous firing frequency of DRn 5-HT neurons recorded in PE rats in the absence (*n* = 38, *N* = 10 from 5 litters) and presence of DNQX and AP5 (*n* = 20, *N* = 7 from 3 litters, Mann-Whitney test: *p* < 0.01). *n* = number of cells, *N* = number of rats.

Serotonin neuron of the DRn are clustered in various groups, including the dmDRn and vmDRn subdivisions, which project extensively to medial prefrontal cortex (mPFC) and central amygdala (CeA), and play a key role in regulating anxiety-like behaviors [32, 33]. Therefore, we examined whether PE alters the function of dmDRn and vmDRn 5-HT neurons. To that end, we first conducted cell-attached recordings, which revealed that in control rats, dmDRn and vmDRN 5-HT neurons were not spontaneously active (0.01 ± 0.005 Hz; Fig. 2A_1_-A_2_), which was consistent with previous reports [28, 34]. In contrast, in PE rats, most dmDRn and vmDRN 5-HT neurons exhibited spontaneous firing activity (0.31 ± 0.05 Hz; Fig. 2A_1_-A_2_), demonstrating that PE increases the excitability of adult DRn 5-HT neurons. We next assessed the impact of PE on the intrinsic electrical properties DRn 5-HT neurons and found that PE had no effects on cell input resistance (PC: 1.30 ± 0.12 GΩ; PE: 1.05 ± 0.11 GΩ; *n* = 8; t-test, t _(7)_ = −1.52, *p* > 0.05), membrane capacitance and evoked firing activity. These results indicate that the PE-induced increase in the excitability of DRn 5-HT neurons is not mediated by alterations of their intrinsic electric properties.

### Prenatal ethanol exposure potentiates glutamate synapses of DRn 5-HT neurons

The firing activity of DRn 5-HT neurons is tightly regulated by the strength of their excitatory inputs [35, 36]. Consequently, the increased activity of DRn 5-HT neurons in PE rats could be mediated by a potentiation of DRn glutamate synapses. We first tested this possibility by examining whether blockade of glutamatergic transmission can reverse the increased activity of 5-HT neurons in PE rats. We found that administration of DNQX (20 µM) and AP-5 (20 µM), antagonists of α-amino-3-hydroxy-5-methyl-4-isoxazole propionic acid receptor (AMPAR) and N-methyl-D-aspartate receptor (NMDAR), respectively, significantly reduced the PE-induced activation of 5-HT neurons (PE: 0.37 ± 0.04 Hz; PE + DNQX & AP5: 0.16 ± 0.02 Hz; Fig. 2B_1_-B_2_). Such a finding indicates that a persistent potentiation of DRn glutamate synapses contributes to the PE-induced activation of 5-HT neurons.

**Fig. 3:**
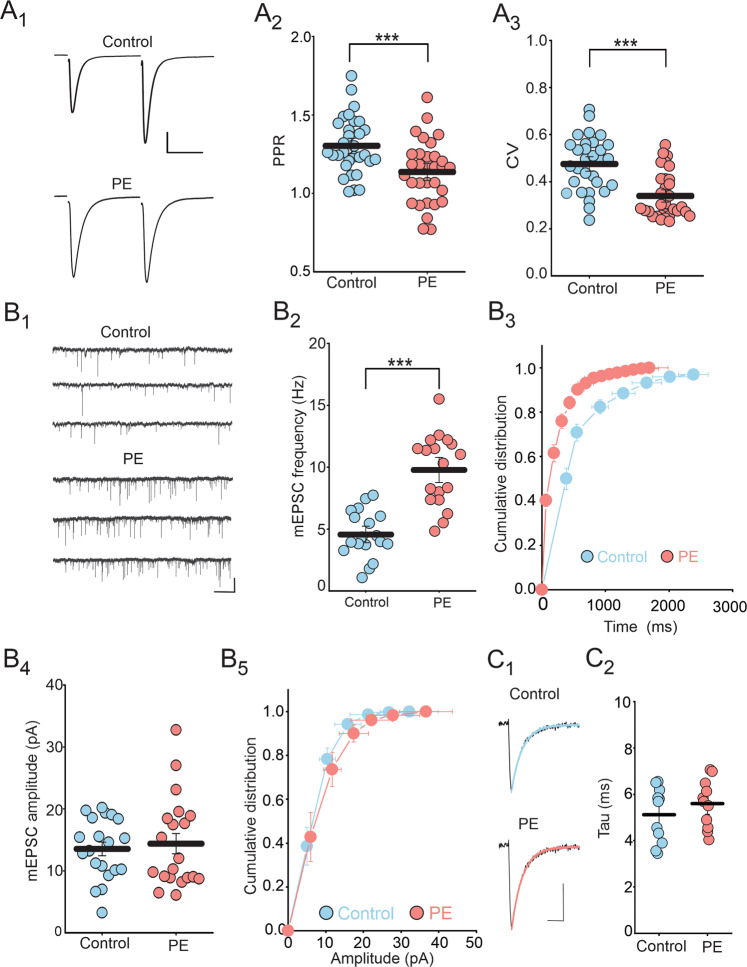
Prenatal ethanol exposure increases glutamate release in the DRn. **A** PE decreases both the PPR and CV of EPSCs (**A**_**1**_) Representative pairs of EPSC traces (average of 60 consecutive trials) evoked by paired stimuli (50 ms interval) in control (left trace) and in PE rats (right trace). (**A**_**2**_) Summary plot of the PPR magnitude of EPSCs obtained in control (, *n* = 31, *N* = 11 from 5 litters) and in PE rats (, *n* = 31, *N* = 11 from 5 litters, independent-samples t-test: *p* < 0.001). **A**_**3**_ Summary plot of the CV of EPSCs in control (, *n* = 32, *N* = 11 from 5 litters) and PE rats (, *n* = 32, *N* = 11 from 5 litters, Mann-Whitney test: *p* < 0.001). (**B**) PE increases the frequency, but not the amplitude of mEPSCs. **B**_**1**_ Representative current traces recorded from control (upper traces) and PE rats (lower traces). **B**_**2**_ Scatter plots of the average mEPSC frequency recorded in control (, *n* = 18, *N* = 6 from 3 litters) and PE rats (, *n* = 18, *N* = 6 from 3 litters, independent-samples t-tests: *p* < 0.001). **B**_**3**_ Cumulative distribution of mEPSC frequency obtained in control and PE rats (*n* = 18, *N* = 6 from 3 litters, K-S test: *p* < 0.05). **B**_**4**_ Scatter plots of the average mEPSC amplitude recorded in control (, *n* = 20, *N* = 7 from 4 litters) and PE rats (, *n* = 20*, N* = 7 from 4 litters, independent-samples t-tests: *p* > 0.05). **B**_**5**_ Cumulative distribution of mEPSC amplitude obtained in control () and PE rats () (*n* = 20, *N* = 7 from 4 litters, K-S test, *p* > 0.05). **C** PE has no effect on the decay kinetic of mEPSCs. **C**_**1**_ Averaged mEPSC traces (100) fitted with single exponential decay function and recorded from control (upper traces) and PE rats (lower traces). Scale bars: 5 pA, 5 ms. **C**_**2**_ Averaged decay time constant of mEPSCs obtained in control () and PE rats () (*n* = 11, *N* = 5 from 3 litters, independent-samples t-tests: *p* > 0.05). *n* = number of cells, *N* = number of rats.

Next, to directly examine the impact of PE on glutamate synapses, we assessed the probability of glutamate release using the paired-pulse ratio (PPR) and coefficient of variation (CV) of eEPSCs. Compared to control, PE significantly reduced the PPR (Control: 1.30 ± 0.03; PE: 1.13 ± 0.02; Fig. 3A_1_-A_2_) and CV of eEPSCs (Control: 0.47 ± 0.02; PE: 0.33 ± 0.01; Fig. 3A_3_), thereby indicating persistent potentiation of glutamate release in the DRn. Consistent with this conclusion, we also found that compared to control rats, PE enhanced the average mEPSC frequency (Control: 4.56 ± 0.44 Hz; PE: 9.77 ± 0.67 Hz; Fig. 3B_1_-B_2_) as indicated by the leftward shift of the cumulative distribution of mEPSC frequency (K-S test: *p* < 0.05, Fig. 3B_3_). In contrast, PE did not affect the average amplitude (Control: 13.53 ± 1.09 pA; PE: 14.38 ± 1.63 pA; Fig. 3B_4_), the cumulative distribution (K-S test: *p* > 0.05, Fig. 3B_5_) and decay kinetic of mEPSCs (Tau control: 5.12 ± 0.36 ms; Tau PE: 5.59 ± 0.31 ms; Fig. 3C_1_-C_2_), indicating the PE did not alter the function of postsynaptic AMPARs. Taken together, these results indicate that the PE-induced increased electrical activity of 5-HT neurons, is in large part, mediated by a persistent potentiation of glutamate release in the DRn.

### Prenatal ethanol exposure enhances tonic NO signaling at DRn glutamate synapses

**Fig. 4:**
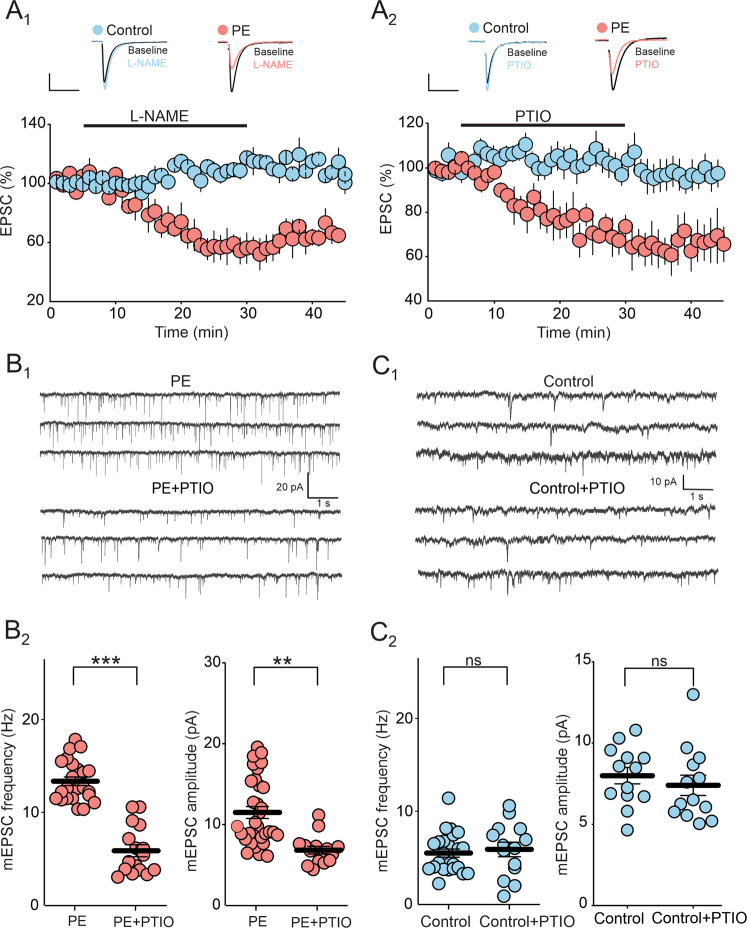
Increased tonic NO signaling contributes to PE-induced potentiation of DRn glutamate synapses. **A** Inhibition of tonic NO signaling depresses the amplitude of EPSCs in PE, but not in control rats. **A**_**1**_ The NOS inhibitor L-NAME inhibits the amplitude of EPSCs only in PE rats. Lower panel is a time-course summary of normalized EPSC amplitude depicting the depression of EPSC amplitude induced by L-NAME (100 µM) in PE (, *n* = 8, *N* = 4 from 2 litters, paired t-test: *p* < 0.01, vs baseline), but not in control rats (, *n* = 9, *N* = 4 from 2 litters, paired t-test: p > 0.05, vs baseline). Upper panel illustrates representative EPSC traces taken before (1) and during L-NAME application (2). Scale bars: 25 pA, 10 ms. **A**_**2**_ The NO scavenger PTIO depresses the amplitude of EPSCs in PE, but not in control rats. Lower panel depicts the depression of EPSC amplitude induced by PTIO (100 µM) in PE (, *n* = 9, *N* = 4 from 2 litters, paired t-test: *p* < 0.05, vs baseline) compared to control (, *n* = 8, *N* = 4 from 2 litters, paired t-test: *p* > 0.05, vs baseline). Upper panel represents sample EPSC traces collected before (1) and during PTIO application (2). Scale bars: 25 pA, 10 ms. **B** Inhibition of tonic NO signaling reduces the frequency and the amplitude of mEPSCs in PE rats. **B**_**1**_ Upper and lower graphs illustrate sample current traces recorded from PE rats in the absence and presence of PTIO (100 µM), respectively. Scale bars: 500 ms, 10 pA. **B**_**2**_ Summary of the effects of PTIO on the frequency and amplitude of mEPSC in PE rats. Left graph represents scatter plots of mEPSC frequency recorded in PE rats in the absence (PE, *n* = 23, *N* = 8 from 4 litters) and presence of PTIO (PE + PTIO, *n* = 16, *N* = 5 from 3 litters, Mann-Whitney test: *p* < 0.001, PE vs PE + PTIO). Right panel depicts scatter plots of the average mEPSC amplitude recorded in PE rats in the absence (PE, *n* = 32, *N* = 10 from 5 litters) and presence of PTIO (PE + PTIO, *n* = 15, *N* = 5 from 3 litters, Mann-Whitney test: *p* < 0.01 PE vs PE + PTIO). (**C**) Inhibition of tonic NO signaling has no effect on the frequency and amplitude of mEPSCs in control rats. (**C**_**1**_) Sample current traces recorded from control rats in the absence and presence of PTIO (100 µM), respectively. (**C**_**2**_) Left panel illustrates the frequency of mEPSC recorded in the absence (*n* = 24, *N* = 8 from 4 litters) and presence of PTIO (*n* = 14, *N* = 5 from 3 litters, paired t-test: *p* > 0.05). Right panel depicts the amplitude of mEPSC recorded in the absence (*n* = 33, *N* = 11 from 6 litters) and presence of PTIO (*n* = 13, *N* = 6 from 3 litters, paired t-test: *p* > 0.05). *n* = number of cells, *N* = number of rats.

The strength of DRn glutamate synapses is regulated by various signaling molecules, including nitric oxide (NO) and endocannabinoids (eCBs) signaling. We have previously shown that NO and eCBs retrogradely control the strength of DRn glutamate synapses [37, 38]. Thus an increase in NO and eCBs signaling induce long-term potentiation and depression of glutamate synapses in the DRn, respectively [37–39]. Because PE alters the function of neuronal nitric oxide synthase (nNOS) function [40] and enhances the formation of reactive nitrogen species (RNS), including peroxynitrite (ONOO^-^) [41, 42], we hypothesized that an enhanced NO signaling could mediate the PE-induced potentiation of DRn glutamate synapses. We first tested this hypothesis by examining whether reducing tonic NO signaling using NOS inhibitors and NO scavengers can reverse the potentiation of glutamate synapses in PE rats. Remarkably, inhibition of NOS with L-NAME (100 µM) significantly reduced the amplitude of eEPSCs only in DRn slices of PE rats (Control: 108.2 ± 1.98 % of baseline; PE: 58.21 ± 5.5 % of baseline; *p* < 0.05, ɳ^2^_P_ = 0.73, PE vs PC; Fig. 4A_1_). Similarly, bath application of the NO scavenger PTIO (100 µM) significantly reduced the amplitude of eEPSCs in PE, but not control rats (Control: 102.36 ± 7.67% of baseline; PE: 70.85 ± 5.92 % of baseline; *p* < 0.05, ɳ^2^_P_ = 0.42, PE vs PC; Fig. 4A_2_). These observations suggest that an increase in tonic NO signaling contributes to the PE-induced potentiation of DRn glutamate synapses. Consistent with this notion, reducing NO function in the DRn with PTIO (100 µM) inhibited the frequency and the amplitude of mEPSCs in PE (PE: 13.53 ± 0.55 Hz; PE + PTIO: 5.85 ± 0.65; Fig. 4B_1_-B_2_), but not in control rats (Control: 5.68 ± 0.41 Hz; Control + PTIO: 5.88 ± 0.78 Hz; Fig. 4C_1_-C_2_). Collectively, these results indicate that an enhanced tonic NO signaling mediates the PE-induced potentiation of DRn glutamate synapses.

**Fig. 5:**
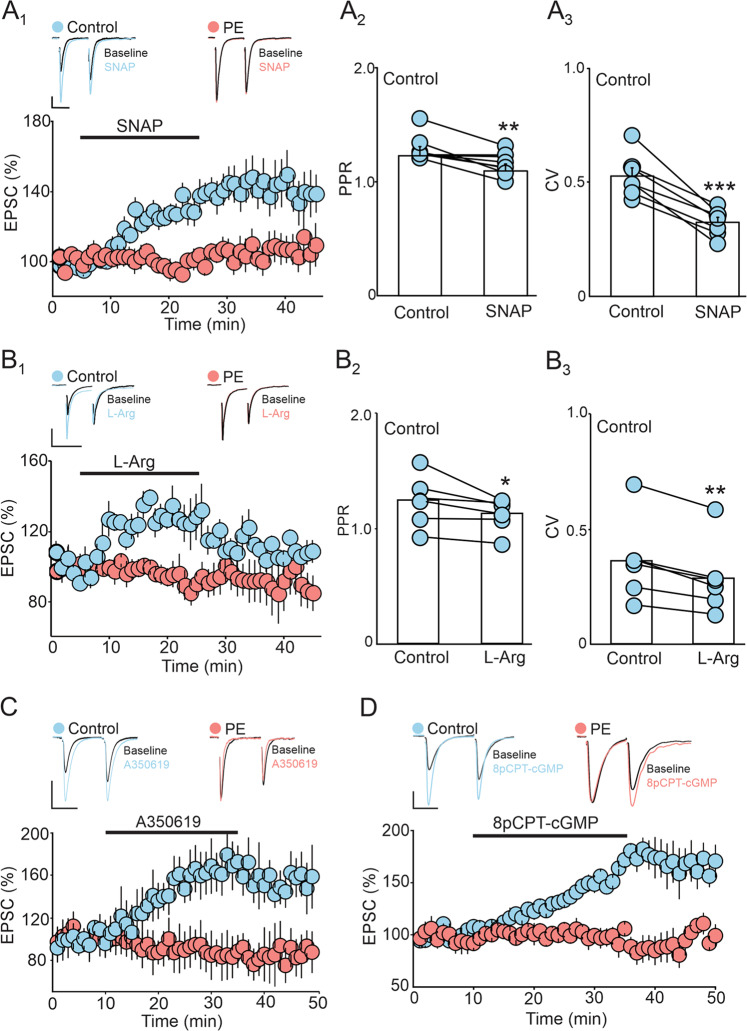
Prenatal ethanol exposure occludes the potentiation of DRn glutamate synapses induced by activation of NO/sGC/PKG pathway. **A** The potentiation of EPSCs amplitude induced by the NOS activator SNAP is blunted in PE rats. **A**_**1**_ Lower graph depicts a time-course summary of the potentiation of EPSC amplitude induced by SNAP (200 µM) in control (, *n* = 13, *N* = 6 from 3 litters, paired t-test: *p* < 0.01, vs baseline) and PE rats (, *n* = 11, *N* = 5 from 3 litters, paired t-test: *p* > 0.05, vs baseline). Upper panel illustrates representative EPSC traces taken before (1) and during SNAP application (2). Scale bars: 50pA, 10 ms. (**A**_**2**_ – **A**_**3**_) Summary graphs of the effect of SNAP (100 µM) on PPR (*n* = 11, *N* = 5 from 3 litters, paired t-test: *p* < 0.01, vs control) and CV (*n* = 11, *N* = 5 from 3 litters, paired t-test: *p* < 0.001, vs control) in control rats. Note that the SNAP-induced potentiation of EPSC amplitude in control rats is associated with reduction of both PPR and CV. (**B**) PE blocks the potentiation of EPSC amplitude induced by the NO precursor L-Arg. (**B**_**1**_) Lower panel illustrates a time-course summary of the potentiation of EPSC amplitude induce by L-Arg (100 µM) in control (, *n* = 6, *N* = 4 from 2 litters, paired t-test: *p* < 0.01, vs baseline) and PE (, *n* = 8, *N* = 4 from 2 litters, paired t-test: *p* > 0.05, vs baseline). Upper graph depicts superimposed sample EPSC traces collected before (1) and during L-Arg application (2). Scale bars: 50 pA, 20 ms. (**B**_**2**_ **– B**_**3**_) Summary graphs of the effect of L-Arg (100 µM) on PPR (*n* = 7, *N* = 4 from 2 litters, paired t-test: *p* < 0.05) and CV (*n* = 6, *N* = 4 from 2 litters, paired t-test: *p* < 0.01) in control rats. Note that the L-Arg-induced potentiation of EPSC amplitude in control rats is associated with a significant decrease in PPR and CV. (**C**) PE blocks the potentiation of EPSC amplitude induced by sGC activator. Lower panel is a time-course of the effect of sGC activator A350619 (100 µM) on the amplitude of EPSCs in control (, *n* = 8, *N* = 5 from 3 litters, paired t-test: *p* < 0.001, vs baseline) and PE (, *n* = 11, *N* = 5 from 3 litters, paired t-test: *p* > 0.05, vs baseline) rats. Upper graph depicts representative EPSC traces taken before (1) and during A350619 application (2) in control (left traces) and PE rats (right traces). Scale bars: 50 pA, 20 ms. (**D**) PE blocks the potentiation of EPSCs induced by direct activation of PKG. Lower panel is a time-course summary depicting the effect of PKG activator 8pCPT-cGMP (100 µM) on EPSC amplitude in control (, *n* = 7, *N* = 4 from 2 litters, paired t-test: p < 0.001, vs baseline) and PE rats (, *n* = 6, *N* = 4 from 2 litters, paired t-test: *p* > 0.05, vs baseline). Upper graph depicts representative EPSC traces taken before (1) and during 8pCPT-cGMP application (2) in control (left traces) and PE rats (right traces). Scale bars: 50 pA, 20 ms. *n* = number of cells, *N* = number of rats.

In principle, if the PE-induced potentiation of DRn glutamate synapses were to be mediated by an enhanced tonic NO signaling and persistent activation of soluble guanylate cyclase and protein kinase G (sGC/PKG) signaling cascade, then due to an occlusion effect, direct activation of this signaling pathway should not potentiate eEPSC amplitude in PE rats. We tested this possibility by conducting three independent sets of experiments. First, we examined the effect of NOS activation on the amplitude of eEPSCs in control and PE rats. As expected, we found in control rats, administration of NO donor SNAP (200 µM) potentiated the eEPSC amplitude (143.96 ± 10.90 % of baseline; Fig. 5A_1_), and increased glutamate release, as indicated by the decrease in PPR (Baseline: 1.21 ± 0.08; SNAP: 1.11 ± 0.08; Fig. 5A_2_) and CV (Baseline: 0.45 ± 0.04; SNAP: 0.30 ± 0.02; Fig. 5A_3_). In contrast, administration of SNAP (200 µM) did not alter the eEPSC amplitude in PE rats (100.64 ± 3.91 % of baseline; *p* < 0.05, ɳ^2^_P_ = 0.50, PE vs PC; Fig. 5A_1_). Next, we examined the effect of NO precursor L-Arg on eEPSC amplitude in control and PE rats and found that L-Arg increased the eEPSC amplitude in control, but not in PE rats (Control: 129.06 ± 4.29 % of baseline; PE: 108.57 ± 7.77 % of baseline; *p* < 0.05, ɳ^2^_P_ = 0.78, PE vs PC; Fig. 5B_1_). The L-Arg induced potentiation of eEPSC amplitude was mediated by an increase in glutamate release as indicated by the decrease in PPR (Baseline: 1.24 ± 0.09; L-Arg: 1.11 ± 0.06; Fig. 5B_2_) and CV of eEPSCs (Baseline: 0.36 ± 0.07; L-Arg: 0.28 ± 0.06; Fig. 5B_3_). Finally, because activation of sGC/PKG pathway mediate NO-induced potentiation of DRn glutamate synapses, we assessed the impact of PE on the sGC/PKG pathway-mediated potentiation of eEPSC amplitude. We found that only in control rats, activation of sGC with A350619 (100 µM) enhanced the eEPSC amplitude (Control: 162.68 ± 11.86 % of baseline; PE: 90.40 ± 5.41 % of baseline; p < 0.05, ɳ^2^_P_ = 0.68, PE vs PC; Fig. 5C). Similarly, activation of PKG with 8pCPT-cGMP (100 µM), potentiated the amplitude of eEPSCs only in control rats (Control: 156.23 ± 2.87 % of baseline; PE: 98.13 ± 5.86 % of baseline; *p* < 0.05, ɳ^2^_P_ = 0.88, PE vs PC; Fig. 5D). Together these results support the hypothesis that a persistent activation of NO/sGC/PKG signaling pathway contribute to the PE-induced potentiation of DRn glutamate synapses.

### Prenatal ethanol impairs tonic eCB signaling at DRn glutamate synapses

**Fig. 6:**
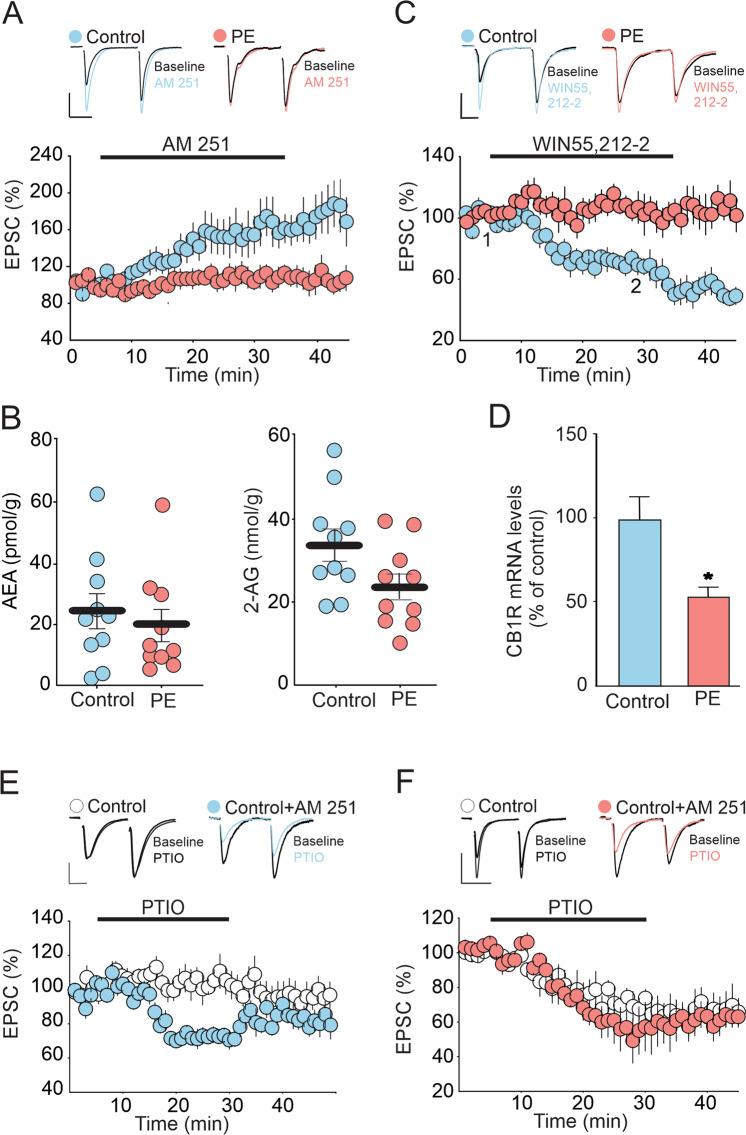
PE impairs tonic eCB signaling at DRn glutamate synapses by downregulating CB1 receptors. **A** PE abolishes tonic eCB-mediated inhibition of glutamatergic synaptic transmission in the DRn. Lower panel is a summary of the time course of normalized EPSCs illustrating the potentiation of EPSC amplitude induced by CB1 antagonist AM 251 (3 µM) in control (, *n* = 10, *N* = 5 from 3 litters, paired t-test: *p* < 0.01, vs baseline) and in PE rats (, *n* = 9, *N* = 5 from 3 litters, paired t-test: *p* > 0.05, vs baseline). Upper panel illustrates superimposed averaged EPSC traces collected before (1) and during application (2) of AM 251 (3 µM). Scale bars: 50 pA, 20 ms. (**B**) PE does not alter the levels of AEA and 2-AG in the DRn. Left panel depicts summary of the average AEA levels in the DRn of control (, *n* = 9, *N* = 5 from 3 litters) and PE (, *n* = 9, *N* = 5 from 3 litters, independent t-test: *p* > 0.05, vs PE). Right panel illustrates a summary of the average 2-AG levels in the DRn of control (, *n* = 9, *N* = 5 from 3 litters) and PE (, *n* = 9, *N* = 5 from 3 litters, independent t-test: *p* > 0.05, vs PE). (**C**) PE blocks the inhibition of EPSC amplitude induced by the CB1R agonist win 55,212-2. Lower graph is a time course summary of the effects of Win 55,212-2 (10 µM) on EPSC amplitude in control (, *n* = 11, *N* = 6 from 3 litters, paired t-test: *p* < 0.05, vs baseline) and PE rats (, *n* = 8, *N* = 5 from 3 litters, paired t-test: p > 0.05, vs baseline). (**D**) PE reduces the mRNA levels of CB1R in the DRN (independent t-test: p < 0.05, control vs PE). (**E**) In control rats, persistent blockade of CB1R enhances tonic NO signaling which mimics the effect observed in PE rats. Lower graph is time course summary of the effect of NO scavenger PTIO (100 µM) on the amplitude of EPSCs obtained in DRn slices pre-incubated without (, *n* = 9, *N* = 4 from 2 litters, paired t-test: *p* > 0.05, vs baseline) and with AM 251 (3 µM) (, *n* = 9, *N* = 5 from 3 litters, paired t-test: *p* < 0.001, vs baseline) in control rats. Upper graph is representative EPSCs traces taken before (1) and during PTIO application (2) in without (left traces) and with AM 251(right traces) pre-incubation. Scale bars: 50 pA, 20 ms. (**F**) In PE rats, persistent blockade of CB1R does not alter the depression of EPSC amplitude induced by NO scavenger PTIO. Lower panel is a summary of the time course of the depression of EPSCs induced by PTIO (100 µM) in DRn slices pre-incubated without (, *n* = 9, *N* = 5 from 3 litters, paired t-test: *p* < 0.001, vs baseline) and with AM 251 (3 µM) (, *n* = 8. *N* = 4 from 2 litters, paired t-test: *p* < 0.001, vs baseline) in PE rats. Upper graph is representative EPSCs traces taken before (1) and during PTIO application (2) in slices pre-incubated without (left traces) and with AM 251 (right traces), respectively. Scale bars: 100 pA, 25 ms. *n* = number of cells, *N* = number of rats.

Glutamate synapses in the DRn are also controlled by tonic eCB signaling [38]. Manipulations that increase and decrease tonic eCB signaling depress and potentiate the strength of these synapses, respectively [38]. Thus, it is possible that an impaired eCB signaling in PE rats could also contribute to the potentiation of DRn glutamate synapses. We examined this possibility by assessing the effects of PE on tonic eCB signaling measured by the blockade of CB1R-induced potentiation of EPSC amplitude [38]. Consistent with a previous report [38], in control rats, blockade of CB1Rs with AM 251 (3 µM) potentiated the eEPSC amplitude and increased glutamate release (163.81 ± 9. 2 % of baseline; Fig. 6A), indicating the presence of a tonic eCB signaling in the DRn. In contrast, in PE rats, the AM 251-induced potentiation of eEPSC amplitude was blunted (103. 9 ± 7.25 % of baseline; *p* < 0.05, ɳ^2^_P_ = 0.54, PE vs PC; Fig. 6A), indicating that PE impaired tonic eCB signaling. Mechanistically, PE could impair tonic eCB signaling by inhibiting eCB synthesis and release, reducing CB1R function, or a combination of both. To test these possibilities, we first measure the levels of anandamide (AEA) and 2-arachydonyl glycerol (2-AG) two major eCBs (i.e. AEA and 2-AG) in the DRn and found that PE did not significantly alter AEA (Control: 24.03 ± 5. 73 pmol/g; PE: 19.34 ± 5.25 pmol/g; Fig. 6B) and 2-AG levels (Control: 33.85 ± 3.93 nmol/g; PE: 23.699 ± 3.17 nmol/g; Fig. 6B), indicating that the impaired tonic eCB signaling in PE rats was not mediated by a deficit in AEA and 2-AG synthesis.

Next, we tested the effect of PE on the function and expression of CB1R. To that end, we examined the impact of PE on the depression of EPSC amplitude induced by the CB1R agonist WIN 55, 212-2 (WIN, 10 µM). We found that WIN55, 212-2 depressed the eEPSC amplitude in control (76.26 ± 7.5 % of baseline; Fig. 6C), but had no effect in PE rats (106.05 ± 10.12 % of baseline; *p* < 0.05, ɳ^2^_P_ = 0.53, PE vs PC; Fig. 6C), indicating an impaired CB1R function. We also assessed the expression of CB1Rs in the DRn using qPCR and found that compared to control, PE significantly reduced the levels of CB1R mRNA (53.65 ± 5.68 % of control; Fig. 6D). Collectively, these results indicate that PE impairs tonic eCB signaling by reducing CB1R function.

The findings that PE persistently enhances NO function while impairing tonic eCB signaling raised the possibility of a functional cross talk between these two systems. Specifically, because nNOS activity is regulated by baseline network activity, we hypothesized that the PE-induced blockade of tonic eCB function, which increases neuronal activity, may contribute to enhanced NO signaling in the DRn of PE rats. We tested this possibility by examining whether in control rats, blocking tonic eCB with the CB1R antagonist/reverse agonist AM 251, could mimic the increased NO signaling observed in PE rats. Tonic NO signaling was assessed by the depression of eEPSC amplitude induced by NO scavenger. We found that incubating DRn slices from control rats with AM 251 (3 µM) significantly increased the depression of eEPSCs induced by the NO scavenger PTIO (100 µM) (In control: 103.25 ± 1.46; In AM 251: 72.22 ± 2.07 % of baseline; *p* < 0.05, ɳ^2^_P_ = 0.95, Control vs Control+AM251; Fig. 6E), thereby suggesting that blocking eCB function enhances tonic NO signaling. In contrast, in PE rats, which already exhibit a strong PTIO-induced depression of eEPSCs, blockade of CB1R with AM 251 did not further enhanced the PTIO- induced depression of eEPSC amplitude (PE control: 62.52 ± 1.4 % of baseline; PE AM 251: 58.38 ± 1.43; *p* > 0.05, ɳ^2^_P_ = 0.19, PE vs PE + AM251; Fig. 6F). Taken together, these results strongly support the hypothesis that an impaired CB1R function contributes, at least in part, to the increased tonic NO signaling in the DRn of PE rats.

## Discussion

While clinical and preclinical studies have revealed strong associations between abnormal 5-HT system and anxiety disorders observed in FASD, the mechanisms by which PE alters the function of 5-HT neurons remain unknown. Here, using a rodent model of FASD, we show that PE increases anxiety-like behaviors in adult male rats and persistently activates DRn 5-HT neurons. Importantly, we report that the hyper-serotonergic phenotype of PE rats is mainly mediated by a potentiation of DRn glutamate synapses. Furthermore, we demonstrate that the PE-induced potentiation of these synapses is caused by enhanced nitrergic function and impaired eCB signaling in the DRn. As such, the present study reveals novel cellular mechanisms by which PE alters the function of 5-HT system, which could contribute to the increased anxiety-like behaviors reported in PE rats.

Prenatal ethanol exposure is detrimental to the normal brain development and a major risk factor for mood disorders, including anxiety-like behaviors. Indeed, results from numerous behavioral studies in mice and rats have shown that ethanol exposure during the gestational period equivalent to second and third trimester of human pregnancy alters stress homeostasis [43, 44] and increases anxiety-like behaviors [12, 13, 45]. Consistent with this notion, our results show that PE induces an anxiety-like phenotype in adult male rats. A novel finding of this study is that PE also induces a persistent activation of DRn 5-HT neurons. Because activation and inhibition of these neurons exert anxiogenic and anxiolytic-like effects, respectively [46, 47], it is possible that persistent activation of these neurons could contribute to increased anxiety-like behaviors of PE rats. It is noteworthy that inhibition of these neurons via activation of 5-HT_1A_ receptors [48] reduces anxiety-like behaviors in animal model of FASD [49]. Previous studies have reported that PE also increases anxiety-like behaviors in female rats [11, 48]. However, it remains unknown whether the anxiety phenotype of female PE rats is associated with a persistent activation of DRn 5-HT neurons. Future studies are necessary to determine the effects PE on the excitability of DRn 5-HT neurons of adult female rats.

The present study reveals that the PE-induced persistent activation of DRn 5-HT neurons is mainly mediated by a potentiation of their glutamatergic inputs. Indeed, blockade of ionotropic glutamate receptors reverses the PE-induced activation of DRn 5-HT neurons. Furthermore, our results show that PE potentiates DRn glutamate synapses by increasing glutamate release as indicated by the decrease in PPR, and CV of EPSCs as well as by the increase of mEPSCs frequency. Interestingly, similar effects have been reported in other brain areas in rats [12, 50] and mice [49], indicating that an increased glutamatergic synaptic transmission may be a common mechanism by which PE persistently enhances neuronal excitability in the brain.

Although, our results establish that PE potentiates DRn glutamate synapses, it remains unknown whether such a potentiation of DRn glutamatergic inputs mediates the increased anxiety-like behaviors in PE rats. It is noteworthy that previous behavioral studies have shown that activation of lateral habenula (LHb) and medial prefrontal cortex (mPFC)-DRn excitatory circuits enhances stress responses [51] and increases anxiety-like behaviors [52]. In addition, recent optogenetic studies have reported that activation of DRn 5-HT neurons projecting to the bed nucleus of the stria terminalis and central amygdala (CeA) promotes anxiety-like behaviors [53, 54]. However, whether PE persistently alters the function of these neuronal circuits remains to be elucidated. Clearly, future optogenetic and behavioral studies are required to dissect the precise excitatory circuit(s) and DRn 5-HT subgroups encoding anxiety-like behaviors in PE rats.

PE is known to increase glutamate release in other brain areas [12, 55, 56], but the mechanisms underlying this effect are unknown. Here, we report that PE potentiates glutamate release in the DRn via an increase in nitrergic and deficit in eCB signaling. Several lines of evidence support this conclusion. First, in PE, but not in control rats, inhibition of NOS or administration of NO scavengers profoundly inhibits DRn glutamate synapses. Second, the PE-induced increase in glutamate release is reversed by NO scavengers. Finally, PE occludes the potentiation of glutamate synapses induced by activation of sGC/PKG signaling cascade. The conclusion that PE potentiates glutamate synapses via enhanced NO signaling is consistent with previous findings that PE increases the expression and activity of nNOS in several brain areas [57, 58]. Given that the nitrergic system plays an ubiquitous role in controlling synaptic transmission and plasticity throughout the brain [37, 59, 60], it is conceivable that an enhanced NO signaling may mediate the effects of PE on glutamate synapses in other brain areas, though future studies are required to test this notion.

In the brain, at low concentration, NO exerts a rapid regulation of synaptic transmission and plasticity via activation of sGC/PKG signaling cascade [61, 62]. NO can also modulate cellular signaling through proteins S-nitrosylation (SNO), a post-translational protein modification (PTM), in which nitrisogroup (-NO) is incorporated into cysteine thiols to form S-nitrosothiols [63, 64]. This later mechanism is believed to mediate the long-term effects of NO on various aspects of neuronal signaling, including maintenance of synaptic plasticity [65, 66]. While our results show that PE potentiates DRn glutamate synapses via activation of NO/sGC/PKG pathways, they do not exclude the possibility that the persistent effect of PE on glutamate release may involve SNO-mediated mechanisms. Interestingly, previous studies have shown that the increase in NO production induces the s-nitrosylation of membrane proteins controlling synaptic vesicles exocytosis, such as syntaxin [67, 68]. The SNO of syntaxin has been shown to mediate the persistent increase in neurotransmitter release observed in animal model of neurodevelopment disorders, such autism spectrum disorders (ASD) [69]. However, whether such a mechanism is also involved in FASD remains to be determined. Future proteomic studies are necessary to determine whether PE increases the s-nitrosylation of membrane proteins involved in the regulation of synaptic transmission and plasticity in the DRn and other brain areas.

The present study also reveals that PE reduces the function of CB1Rs in the DRn, which mediates, at least in part the potentiation of glutamatergic synaptic transmission onto 5-HT neurons. This finding is consistent with earlier reports that PE alters the expression and function of CB1Rs in several other brain areas [70, 71] and supports the general concept that PE persistently impairs eCB function in the brain. Importantly, a novel finding of the present study is that in control, but not PE rats, pharmacological manipulations that reduce tonic eCB mimic the PE-induced increase in NO signaling. A parsimonious interpretation of these findings is that the PE-induced deficit in eCB function leads to enhanced activity and a persistent increase in NO signaling. This in turn mediates the potentiation of glutamate synapses of DRn 5-HT neurons in PE rats. While it remains unknown whether the altered function of nitrergic and eCB systems mediate the anxiety-like behaviors in PE rats, previous behavioral studies have revealed key role of both systems in regulating anxiety-like behaviors. Thus, pharmacological or genetic manipulations that inhibit or activate nNOS, the main NO synthetic enzyme, exerts anxiolytic-like and anxiogenic-like effect [72–75], respectively. Importantly, an increase in NO signaling in the DRn has been shown to mediate anxiety-like behaviors induced by ethanol withdrawal [76] and stress exposure [77]. Similarly, genetic or pharmacological manipulations that reduce eCB signaling exert anxiogenic-like effects [78–81]. These studies support the possibility that enhanced nitrergic function and impaired eCB signaling could mediate the increased anxiety-like behaviors in PE rats.

Results from previous studies have reported that PE alters the development of DRn 5-HT neurons and reduces the density of 5-HT projections [17, 18]. While the mechanisms underlying these effects are unknown, it is possible that the “push-pull” effects exerted by PE on NO and eCB signaling, respectively may also contribute to the neurodegenerative effects of PE on the 5-HT system. In this context, it is well established that excessive activation of NO signaling enhances the production of reactive nitrogen species (RNS), such as peroxynitrite (ONOO-) and induces oxidative stress, which promotes cell death [82–85]. In contrast, eCB signaling is known to exert neuroprotective and anti-inflammatory effects [86–88]. However, whether a deficit in eCB signaling combined with enhanced nitrinergic function contribute to the neurodegenerative effects of PE on the 5-HT system [17, 18] remains to be established. Future anatomical and pharmacological studies are required to directly test this notion.
